# Identifying Predictors of Suicide in Severe Mental Illness: A Feasibility Study of a Clinical Prediction Rule (Oxford Mental Illness and Suicide Tool or OxMIS)

**DOI:** 10.3389/fpsyt.2020.00268

**Published:** 2020-04-15

**Authors:** Morwenna Senior, Matthias Burghart, Rongqin Yu, Andrey Kormilitzin, Qiang Liu, Nemanja Vaci, Alejo Nevado-Holgado, Smita Pandit, Jakov Zlodre, Seena Fazel

**Affiliations:** ^1^Department of Psychiatry, University of Oxford, Oxford, United Kingdom; ^2^Oxford Health NHS Foundation Trust, Warneford Hospital, Oxford, United Kingdom

**Keywords:** risk assessment, feasibility, OxMIS, suicide, schizophrenia, bipolar disorder, electronic health records, natural language processing

## Abstract

**Background:**

Oxford Mental Illness and Suicide tool (OxMIS) is a brief, scalable, freely available, structured risk assessment tool to assess suicide risk in patients with severe mental illness (schizophrenia-spectrum disorders or bipolar disorder). OxMIS requires further external validation, but a lack of large-scale cohorts with relevant variables makes this challenging. Electronic health records provide possible data sources for external validation of risk prediction tools. However, they contain large amounts of information within free-text that is not readily extractable. In this study, we examined the feasibility of identifying suicide predictors needed to validate OxMIS in routinely collected electronic health records.

**Methods:**

In study 1, we manually reviewed electronic health records of 57 patients with severe mental illness to calculate OxMIS risk scores. In study 2, we examined the feasibility of using natural language processing to scale up this process. We used anonymized free-text documents from the Clinical Record Interactive Search database to train a named entity recognition model, a machine learning technique which recognizes concepts in free-text. The model identified eight concepts relevant for suicide risk assessment: medication (antidepressant/antipsychotic treatment), violence, education, self-harm, benefits receipt, drug/alcohol use disorder, suicide, and psychiatric admission. We assessed model performance in terms of precision (similar to positive predictive value), recall (similar to sensitivity) and F1 statistic (an overall performance measure).

**Results:**

In study 1, we estimated suicide risk for all patients using the OxMIS calculator, giving a range of 12 month risk estimates from 0.1-3.4%. For 13 out of 17 predictors, there was no missing information in electronic health records. For the remaining 4 predictors missingness ranged from 7-26%; to account for these missing variables, it was possible for OxMIS to estimate suicide risk using a range of scores. In study 2, the named entity recognition model had an overall precision of 0.77, recall of 0.90 and F1 score of 0.83. The concept with the best precision and recall was medication (precision 0.84, recall 0.96) and the weakest were suicide (precision 0.37), and drug/alcohol use disorder (recall 0.61).

**Conclusions:**

It is feasible to estimate suicide risk with the OxMIS tool using predictors identified in routine clinical records. Predictors could be extracted using natural language processing. However, electronic health records differ from other data sources, particularly for family history variables, which creates methodological challenges.

## Introduction

Suicide risk assessment is central to the care of patients with severe mental illness. Although risk assessment is a routine part of clinical practice, the way in which it is carried out is inconsistent, with no widely accepted standard of care (1, 2). A large number of structured suicide risk assessment tools are used by clinicians (1, 3). The quality of these tools is variable: many have low predictive accuracy and lack scalability. Most commonly used tools have been developed without validated methods (2, 4–6), including pre-specification of risk factors and outcomes, sufficient statistical power (at least 10 outcome events per predictor in derivation studies and 100 outcome events in validation studies), and using multivariable regression to test the incremental value of individual risk factors (6).

One such clinical prediction tool (Oxford Mental Illness and Suicide tool or OxMIS) has been recently developed using high quality methods for individuals with schizophrenia-spectrum and bipolar disorders, who have a high relative risk of suicide (7–9). The tool was developed based on a national cohort of Swedish individuals aged 15–65 with a diagnosis of schizophrenia-spectrum and/or bipolar disorders (involving 58,771 patients, 494 suicides). It includes 17 items, most of them routinely collected. OxMIS has showed good calibration and moderately good discrimination, and has been translated into an online calculator (https://oxrisk.com/oxmis/). OxMIS requires minimal training to use, is scalable and freely available to use with translations into several languages (7). The model formulae and coefficients have been made available with the online calculator.

One challenge for OxMIS and more generally for scalable approaches to clinical assessment and prognosis is external validation outside of the study population in which it was developed. Despite its importance, external validation of risk prediction models is infrequent (10–12). This may lead to overestimation of model accuracy since external validation tends to show poorer predictive performance (10). External validation of suicide risk assessment tools is particularly difficult because, as a rare outcome, large sample sizes are required. Carrying out this kind of external validation prospectively alongside tool implementation is important but requires substantial resources over long periods. The alternative is to use existing epidemiological or clinical data to assess performance of the tool using a retrospective design. Thus, registry data and established cohorts provide possible data sources for validation. However, these may not contain all variables used in a particular diagnostic or prognostic model and are available for a limited selection of populations.

A promising alternative to healthcare registers and existing cohorts is electronic health records (EHRs) (13, 14). There are several potential advantages to using EHRs: they contain large amounts of data on multiple variables, they are available for real-world populations not mirrored in established cohorts, and they can provide information on large samples with sufficient power for rare outcomes (14). On the other hand, EHRs contain information collected primarily for clinical use. One important consideration is that much of the information within them is contained within free-text clinical notes rather than structured fields. This makes extraction of data difficult, relying either on resource-intensive manual review of clinical records, or, increasingly, automated natural language processing (NLP) algorithms (15, 16). NLP processes have been applied to extract a variety of information from free-text clinical records including medication use (17, 18), self-harm (19, 20), and socio-demographic history (21, 22); such approaches have addressed model development using limited numbers of annotated text examples (23). One approach to NLP for EHRs is to build information retrieval systems based on named entity recognition (NER), where a model is trained to recognize concepts which are related to variables of interest. These methods have the potential to widen the range of variables available on a large scale within EHR databases, thus creating new opportunities for their use in suicide research. Previously, EHRs have been used to retrospectively complete risk scores for cardiovascular outcomes, using a score originally developed in an epidemiological cohort (24). However, it remains uncertain whether it is feasible to use electronic health records for external validation of suicide prediction models.

This study aimed to examine whether it is feasible to use routinely collected EHRs for external validation of OxMIS. We approached this feasibility study in two stages: In study 1, we assessed whether the suicide predictors used in the OxMIS tool are present within a particular EHR system, and whether these can be manually extracted in order to calculate risk estimates. In study 2, we addressed the question of whether this process could be scaled up to a larger population using natural language processing in free-text clinical records. To assess the feasibility of this approach, we designed and evaluated a named entity recognition model to extract concepts related to suicide predictors used in OxMIS.

## Methods

### Setting and Data Sources

Oxford Health NHS Foundation Trust provides mental health services to a population of approximately 1.2 million people of all ages in Oxfordshire and Buckinghamshire, UK. This health system uses fully electronic health records, which cover inpatient, outpatient and liaison encounters. The Clinical Records Interactive Search (CRIS) system is a research database containing a de-identified version of Oxford Health’s electronic health record. It contains information on around 170,000 patients, with all EHR records since 2015 and records integrated from preceding years. This database includes structured data fields, for example diagnostic codes, age, gender, and details of previous encounters with mental health services, which are relatively easy to extract for large numbers of individuals. However, much of the information within the health records is contained in free-text documents such as clinical notes and correspondence between clinicians. While structured fields can be directly used, the information held in free-text needs to be extracted with specialized natural language processing (NLP) algorithms or labor-intensive manual review.

For study 1, MB accessed the full, identifiable electronic health record for 57 patients. For study 2, we used de-identified data from the CRIS database.

### Study Population

Our population of interest was individuals with a diagnosis of severe mental illness. We included schizophrenia-spectrum disorders (ICD-8: 295, 297–299; ICD-9: 295, 297–299 excl. 299A; ICD-10: F20–F29) and bipolar disorder (ICD-8: 296 excluding 296.2; ICD-9: 296 excluding 296D; ICD-10: F30–F31). Diagnoses came from ICD codes entered into the electronic health record during routine care.

### Predictive Variables in OxMIS

The extracted risk factors were those used in the OxMIS suicide risk assessment tool^1^ (7). OxMIS provides an estimate of suicide risk in 1 year based on 17 patient variables. During development of the OxMIS tool, 17 variables were selected from routinely collected socio-demographic and clinical risk factors contained in Swedish population-based registers. The variables are: calendar age, sex, previous drug abuse, previous alcohol abuse, previous self-harm, recent antipsychotic treatment, recent antidepressant treatment, current episode (inpatient vs. outpatient), length of first inpatient stay, number of previous episodes, presence of comorbid schizophrenia and depression, highest education, receipt of welfare or disability benefit, previous conviction of a violent offence, parental psychiatric hospitalization, parental drug or alcohol use disorder, and parental suicide. Further details about these variables and the development of OxMIS are reported elsewhere (7).

### Study 1: Calculating Suicide Risk Scores Using Electronic Health Records

To examine whether OxMIS variables can be manually extracted to calculate risk estimates, JZ and SP identified a cohort of 120 patients with schizophrenia-spectrum disorders or bipolar disorder, with currently active inpatient or outpatient encounters at Oxford Health NHS Foundation Trust. Afterwards, MB randomly selected 57 of these individuals and accessed their EHR. Variables were extracted and used to estimate each patient’s risk of suicide at 1 year with the OxMIS calculator. We accessed the full, identifiable EHR for these patients including all notes written during previous contact with Oxford Health NHS Foundation Trust. Data were anonymized after extraction, prior to export outside of the Oxford Health computer system and analysis.

The extracted data were analyzed to describe for each variable: frequency of each variable and proportion of patients with variable information missing from clinical notes. During manual data extraction, some assumptions were made relating to history of parental suicide, parental drug and alcohol use disorder, and parental psychiatric admission. If the notes contained an extensive description of the patient’s family history but did not mention any of these three variables, these were assumed not to be present. Otherwise these were assigned “unknown”.

### Study 2: Developing a Natural Language Processing Model to Extract Variables From Free Text

Our next aim was to investigate the feasibility of scaling up the extraction of OxMIS variables from the EHR to a larger cohort of patients in CRIS. First, the Oxford CRIS team carried out a search to assess whether each predictor is contained within structured fields in CRIS. We identified eight variables with information contained in structured fields. For these, information can be extracted directly for a large cohort. The main focus of study 2 was therefore the remaining nine predictors (**Table 1** shows type of data field for each variable), and how to develop a natural language processing algorithm able to extract these risk factors from free-text documents.

**Table 1:** Study 1 sample characteristics for patients with severe mental illness (n=57).

Variable	Yes (%)	Missing (%)	Type of data field
**Sex (male)**	34 (60%)	0	Structured
**Age: mean (*SD*)**	47 (10.8)	0	Structured
**Previous violent crime**	16 (28%)	0	Free-text
**Previous drug abuse**	18 (32%)	0	Structured
**Previous alcohol abuse**	18 (32%)	0	Structured
**Previous self-harm**	26 (46%)	0	Free-text
**Highest formal education**	Secondary: 26 (46%) Upper-secondary: 10 (18%) Post-secondary: 6 (11%)	15 (26%)	Free-text
**Parental drug/alcohol use disorder**	2 (4%)	4 (7%)	Free-text
**Parental suicide**	1 (2%)	0	Free-text
**Recent antipsychotic treatment**	51 (89%)	0	Free-text
**Recent antidepressant treatment**	19 (33%)	0	Free-text
**Current episode**	Inpatient: 3 (5%) Outpatient: 54 (95%)	0	Structured
**Length of first inpatient stay**	≤7 days: 7 (12%) > 7 days: 50 (88%)	0	Structured
**Number of previous episodes**	≤7: 22 (39%) > 7: 35 (61%)	0	Structured
**Benefits recipient**	31 (54%)	14 (25%)	Free-text
**Parental psychiatric hospitalization**	4 (7%)	9 (16%)	Free-text
**Comorbid depression and schizophrenia**	1 (2%)	0	Structured

Variables identified using manual review of electronic health records. Number of patients (%), unless stated otherwise. Percentages were calculated out of total of 57 patients, including those for whom information was missing.

#### Natural Language Processing Model Design

The task of extracting specific tokens of information from free-text (e.g. violent crime, self-harm, education level) is known as named entity recognition (NER), and extracting our nine risk-factors classifies as such (25). We approached this task using a neural network algorithm, as this method offers higher extraction accuracy and robustness than other techniques (26). The first step in processing for the model is to transform text into a numerical representation (a series of vectors—each being a unique series of numbers representing an individual word) (27). This numerical representation of the text is the input for the neural network model. The characteristics of the model are adapted through a learning process, during which the model is trained using text that has been labelled (annotated) by a researcher (28, 29). Once trained in our dataset, the model was able to identify the location within the text of concepts linked to each of our 9 risk factors (see **Table 1**). This work was based on the Med7 model and implemented using *thinc*^2^ open source python library (30).

In order to address the task of named entity recognition we focused on a set of eight concepts that cover nine OxMIS variables contained within free-text clinical notes. More detailed information can be extracted as “attributes” of the concept—for example the natural language processing model is first trained to identify the broad “concept” of education, and subsequently this is sub-categorized according to an “attribute” describing the level of education (**Supplemental Figure 1**). We have taken an incremental approach to model development. The first stage, which we address in this feasibility study, is identifying the concepts (stage 1 in **Supplemental Figure 1**). If this is feasible, the approach could form the basis of future research refining the model to extract more detailed information on variables. Although this feasibility study primarily focuses on the extraction of broad concepts related to the OxMIS variables, one exception is for medication, where we were able to extract more detailed information. OxMIS considers two medication-related variables: antidepressant use in last 6 months, and antipsychotic use in last 6 months. For annotation and NER model development we combined these variables into one concept: medication use. Once text referring to medications is identified by the NER model, more detailed information about medication types is extracted in the post-processing stage to match the concepts to OxMIS categories. This involves mapping extracted text onto a glossary of terms based on the British National Formulary (BNF) to identify medication groups: antipsychotic, antidepressant, and other.

#### Creating a Training Dataset of Clinical Documents

We first identified a subset of free-text clinical documents to form a training database for our NLP model. In order to maximize the benefits of annotators’ work, we used keyword string matching based on Levenshtein distance to select the most informative documents for the annotators to work on. Specifically, several keywords were predefined for each variable of interest through discussion between authors RY, MS, ANH, QL, and MB based on knowledge of clinical record keeping and relevant literature. We then compared each word in all clinical documents to the predefined keywords and counted the total appearance of these keywords. The documents that returned the highest appearance numbers were taken as the training dataset. The annotation results from these documents were used for model training. The documents used were a mixture of correspondence (for example between psychiatrists and primary care physicians) and free-text notes which are the main day-to-day record of a patient’s interactions with mental health services. We identified 318 of the most informative documents out of all free-text notes from 4,558 patients. Ten documents were used for annotator training and 308 were used to train the NER model.

Next, the training database was annotated according to an annotation schema created by authors AK, QL, NV and MS to be informative for the eight concepts, creating a “gold-standard” annotation training dataset (**Supplemental Figure 1**). We developed the annotation schema through an iterative process using feedback from initial implementation. Afterwards, clinical documents were manually reviewed and annotated according to this schema. The annotation process involved labeling portions of text (spans) which correspond to each concept including sub-labels (attributes) containing auxiliary information about the concepts that are essential to correctly interpret the text, for example whether the concept is negated, and whether it refers to the patient, a parent or another person (**Table 2**, and **Supplementary Figure 1**). The annotation process was carried out using the General Architecture for Text Engineering (GATE) platform (31).

**Table 2:** Summary of annotated electronic health records documents used to train the named entity recognition model.

Variable	Number of annotated text spans	
	Phase 1	Phase 2
History of violence	391	350
History of self-harm	559	397
Formal education	174	200
Medication	1774	3860
Benefits recipient	188	195
Drug/alcohol use disorder	190	130
(Parental) suicide	19	77
Psychiatric admission	332	260
**Total:**	**3,627**	**5,469**

Text spans are words or word combinations that refer to the concept of interest (the variable), as selected by the manual annotator. The model was trained in two phases: first using GATE software and second using Prodigy—an active learning-based annotation tool. The annotated documents shown in this table constituted the “gold-standard” training dataset used in model development. EHR, electronic health record.

#### Training, Refining, and Evaluating the Named Entity Recognition Model

The annotated “gold-standard” training dataset was then used to develop a NER model using deep learning techniques. We trained the model in two phases, in phase 1 we used “gold-standard” annotation data recorded using GATE software, then in phase 2 the model was refined through an iterative process of fine-tuning where examples of the NER model’s output were reviewed and categorized as correct or incorrect. This process was carried out using Prodigy active learning software (32) following metholodogy which we have described in detail elsewhere (33). The NLP model needs to recognize when a concept is mentioned in the text as *not* being present for the patient. For this, negation information was extracted by using the ruled-based NegEx approach, which searches for pre-defined keywords indicating that a concept in text is negated (for example “XX *stopped* taking olanzapine”) (34). We used a modified list of negation keywords to reflect their distribution within CRIS data.

**Figure 1:**
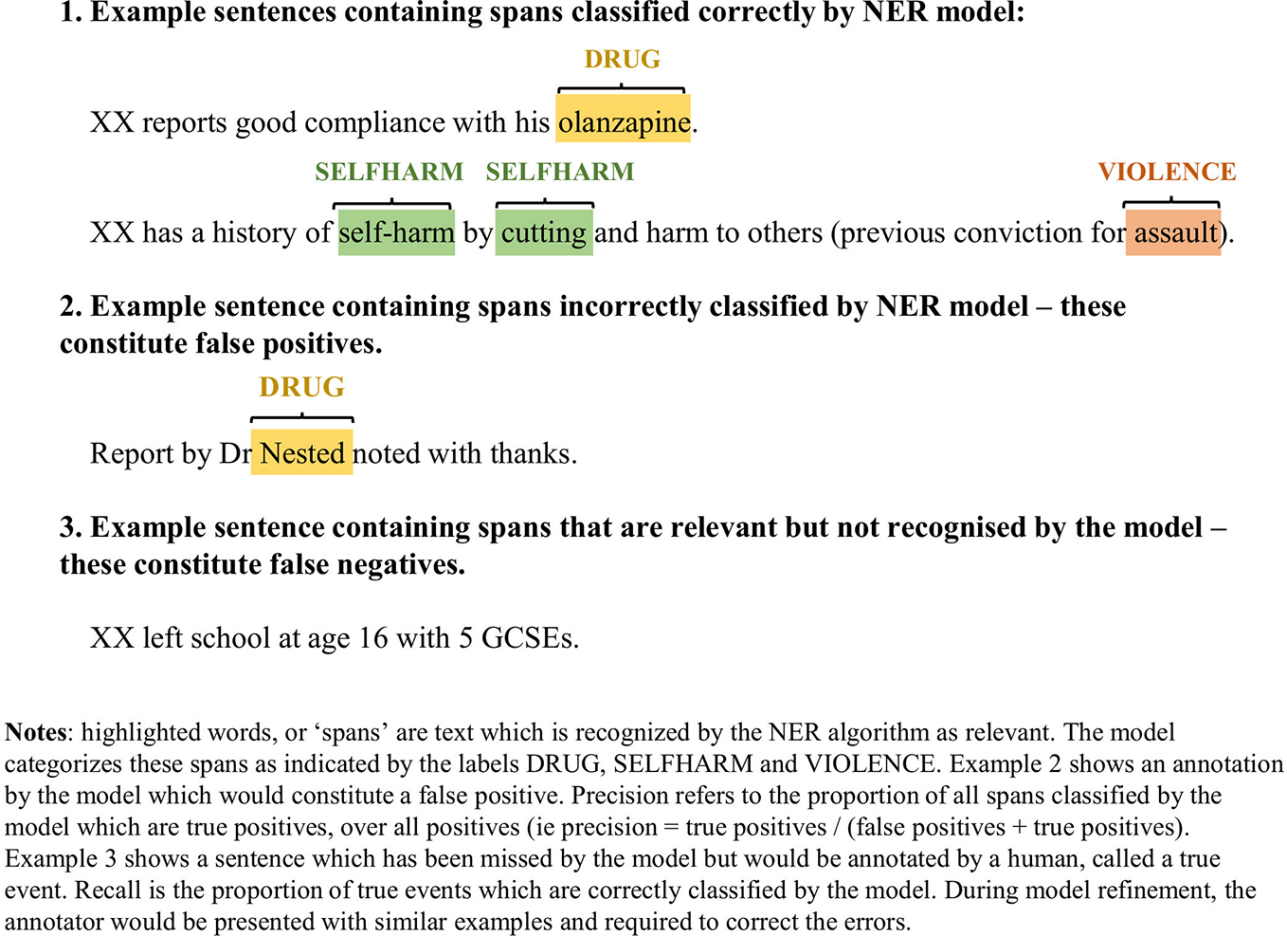
Illustrative examples of sentence classification by named entity recognition model.

We assessed the performance of the NLP model by comparing the results of the model against manual annotations. For each concept of interest, we assessed 1) precision: the fraction of desirable results among all extracted examples. Precision is the proportion of all text spans that the model identifies as belonging to a concept category, which are true positives according to a human annotator. This is similar to positive predictive value; 2) recall: the proportion of true events which are picked up by the model among the total amount of the true events (similar to sensitivity). In this case “true events” refers to text spans which the annotator has categorized as belonging to a concept category; 3) F1 score, which is a harmonic average of precision and recall, giving an overall indication of model performance. **Figure 1** provides illustrative examples of text classification by the NER model.

## Results

### Study 1

Predictive variables were extracted from the clinical notes of 57 patients. Of these patients, 23 were female (40%) with a median age of 48 years, range 23–66 years. Three patients were current inpatients, while 54 were receiving outpatient treatment. OxMIS scores were calculated at the timepoint of accessing the clinical notes.

Of the 17 variables used in OxMIS, 13 were extracted for all patients, with no missing values. For 4 variables, information was not available for a minority: highest formal education, parental drug/alcohol use disorder, receipt of benefits, and parental psychiatric hospitalization. **Table 1** shows each OxMIS variable’s frequency and missing information.

**Figure 2:**
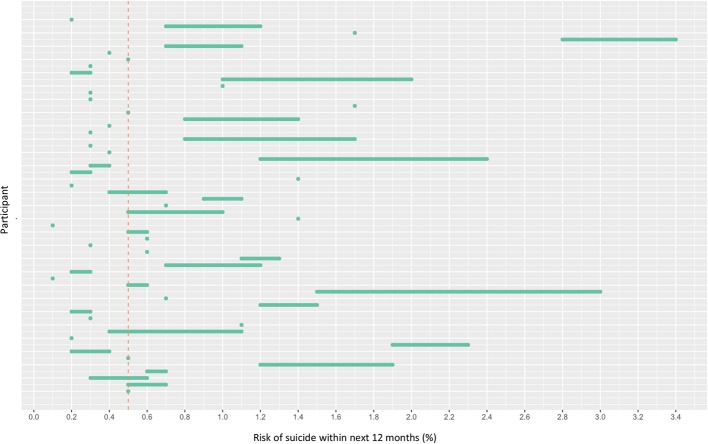
Risk of suicide within 12 months according to OxMIS (Oxford Mental Illness and Suicide tool). Risk was calculated using variables manually extracted from electronic health records. Where variables were unknown, the risk calculator gave a range of risk scores (represented by lines). The line at 0.5 indicates an arbitrary cut-off for an increased risk level.

OxMIS probability risk scores were calculated from clinical records. Where input variables were unavailable or ambiguous, it was possible to estimate risk at 1 year as a range of values incorporating the minimum and maximum options for the missing variable. The median value for estimated suicide risk at one year was 0.5–0.7%, with a range from 0.1% to 3.4% (**Figure 2**). If the cut-off of increased risk was assigned at 0.5% for 1 year, 21 individuals would be categorized as low risk, 33 at increased risk, and three low-increased (with a range of values spanning from below to above 0.5%) (**Figure 2**).

### Study 2

The named entity recognition model was developed in two phases: 1) training with “gold-standard” annotations collected with GATE and 2) model fine-tuning with Prodigy—an active learning-based annotation tool. During the fine-tuning processes with Prodigy, more annotated data was collected. This data was then used for further training of the NER model. The corpus for training and evaluation for phase 1 and phase 2 resulted in a total of 3,627 and 5,469 annotated text spans across eight categories (**Table 2**).

Overall, the NER model achieved (micro averaging) precision of 0.77, recall of 0.90, and F1 of 0.83 on a test dataset (**Table 3**) for extracting concepts related to predictors of suicide in the OxMIS tool. In a test set of annotated text, we used string-matching to identify medication type according to BNF categories. Of 720 text spans correctly identified by the model as referring to medication, 282 referred to antipsychotics and 45 to antidepressants.

**Table 3:** Named entity recognition model performance for concepts related to suicide predictors.

Variable	Manually annotated	Correctly identified	Spurious	Missed	Precision	Recall	F1
**History of violence**	80	60	22	20	0.73	0.75	0.74
**History of self-harm**	90	78	26	12	0.75	0.87	0.80
**Formal education**	29	24	32	5	0.43	0.83	0.56
**Medication**	719	692	128	27	0.84	0.96	0.90
**Benefits recipient**	44	35	15	9	0.70	0.80	0.74
**Drug/alcohol use disorder**	28	17	13	11	0.57	0.61	0.59
**(Parental) suicide**	12	11	19	1	0.37	0.92	0.52
**Psychiatric admission**	53	36	28	17	0.56	0.68	0.62
**Overall micro-average**	1055	953	283	102	0.77	0.90	0.83

Numbers in manually annotated/correctly identified/spurious/missed columns reflect the absolute numbers of text spans related to the concepts in the sample of free-text EHR documents used to assess the model. Spurious results are text spans identified by the model which were not annotated by the researcher (false positives). Micro-averaging figures for overall model performance are based on model performance when text-spans across all concepts are combined. F1 is a measure of overall model performance. EHR, electronic health records.

## Discussion

We examined the feasibility of using routinely collected electronic health records (EHRs) to calculate suicide risk using a scalable clinical prediction model called OxMIS, which generates risk probabilities based on 17 predictors. We tested whether predictors used in OxMIS were present within clinical notes, and if it was feasible to extract them at scale from an anonymized clinical records database using natural language processing.

### Study 1: Manual Calculation of Suicide Risk Using EHRs

In study 1, we assessed the feasibility of calculating OxMIS scores based on manual review of EHRs for 57 patients. We found that OxMIS variables were present in routine clinical notes with few missing variables. Out of 17 variables, four were missing from a minority of patient notes. We were able to calculate suicide probability risk at 1 year for all patients using the OxMIS online tool. For those patients with missing predictors, risk estimates were calculated as a range by the online calculator. We found that estimated suicide risk ranged from 0.1% to 3.4%.

In relation to future implementation of OxMIS in clinical practice, these results suggest that the majority of the included variables are routinely collected as part of clinical evaluation. In addition, our findings suggest that the tool has face validity as it considers factors which are already part of a standard psychiatric history.

### Study 2: Application of Natural Language Processing Tools to Variable Extraction

In study two we assessed the feasibility of scaling-up the extraction of predictors used in the OxMIS tool. This would be necessary in order to use EHRs for external validation in a sufficiently large sample. Eight of the OxMIS variables were present within structured data fields in the EHRs. For the remaining nine predictors, we were able to train a named entity recognition (NER) tool to identify concepts related to each predictor within the electronic health record with good overall accuracy compared to the gold standard of manual evaluation. The overall micro precision was 0.77, recall was 0.90 and F1 was 0.83. This suggests that it is feasible to develop a natural language processing tool to extract these risk factors at scale, using text from the CRIS database. However, we identified challenges which highlight key differences between data contained in EHRs and in population registers. In addition, future research is needed to refine the natural language processing model to extract information which more accurately represents the variables used in the OxMIS tool.

The NER model performed best for extracting information on medication and self-harm, with precision of 0.84 and recall of 0.96 for medication, and precision of 0.75 and recall of 0.87 for self-harm. Performance was poorer for formal education, drug/alcohol use disorder, and parental suicide. Information related to some variables is more difficult to extract using natural language processing. One challenge is that for some variables there are many different ways in which clinicians record information. This includes a variety of related word terms, and also complex and varied linguistic patterns which are evident to a human reader but create challenges when training a NER algorithm. For example, highest formal education may be recorded in the notes in terms of the highest qualification achieved “XX received 5 GCSEs”, the type of institution “XX attended the University of Cardiff from the ages of 18–25” or the age at which the patient left school: “XX left school at 16”. Each example not only includes different words indicating education, but also different linguistic structures. This variation means that an NLP model needs to be trained to recognize several different linguistic patterns which indicate the highest education level. Training the NLP model for these concepts therefore requires more annotated training data than simpler concepts, a factor which may have contributed to poorer model performance for education data.

Another challenge is that some variables are intrinsically mentioned with low frequency in clinical records, as shown in **Tables 2** and **3**. For example, parental drug/alcohol use disorder and suicide was rarely mentioned explicitly. As a result, in study 1, parental drug and alcohol use disorder was found to be one of the missing variables. During manual data extraction, in the absence of explicit documentation, the researcher could infer this information from other text—for example for parental suicide the presence of an extensive family history without mention of suicide, or any mention of parents currently living or who died of other causes would confirm the absence of parental suicide. However, this nuanced approach is difficult to apply during NER model development, where instead model training relies on text spans explicitly referring to the concept. Nevertheless, our NER model performed well considering the limited quantity of annotated examples in the dataset used to train the model for these low-frequency variables. An additional difficulty is where there is a degree of uncertainty in the health record or the variable refers to a complex concept. An example of this arises with the variable: parental drug and alcohol use disorder. For the patient, we can use structured diagnostic codes where clinicians record drug or alcohol use disorder according to clinical definitions. The same approach was used during model development, using population register data on patients and their parents. On the other hand, if the alcohol use of a patient’s parent is mentioned in the EHR, this is rarely recorded in terms of ICD diagnostic categories. Therefore, there is a need to distinguish alcohol or drug use which would not be defined as a disorder from that which would. We found that clinicians tended to record this information with terms such as “heavy drinker”, “alcoholic”, or ambiguous terms such as “XX’s father had problems with drug use”. This creates a challenge when training an NLP algorithm both because there are many different terms used by clinicians to describe drug and alcohol use, and because the distinction between use and misuse is nuanced.

The results presented here show the feasibility of extracting concepts related to the OxMIS predictors using a named entity recognition model trained with free-text from the CRIS database. The results obtained are promising as we have produced a tool with good performance which could be further refined in future research to extract more detailed information on predictors of suicide. For example, if education is mentioned, the level of education needs clarification as a next step. A key challenge for future research is extracting more detailed information may require a large amount of annotated text, at least a few thousand examples for each category, but we found that some concepts were mentioned infrequently in the text. To overcome this challenge, future research will need to expand the database of annotated text which may be facilitated by using CRIS records from different locations. In addition, it will be necessary to incorporate information extraction into a longitudinal timeline representing all information contained within the patient’s EHR. Within the clinical record some historical variables might be mentioned infrequently, but if present on one occasion, they can be carried forward to future time-points.

Previous studies have demonstrated the feasibility of using electronic health record databases to predict suicide attempts and suicide but have some key differences compared to our study. For example, administrative data including medical records have been used to develop a model of suicide risk for active US army soldiers (35), and EHR data have been used to predict post-discharge suicide and suicide attempts in a population of patients discharged from medical care (36). However, these studies have used EHRs in model development, rather than attempting to use EHRs to externally validate a tool developed with register data. In addition, these models have incorporated large numbers of input variables using a data-driven approach which may reduce their generalizability. In contrast, the OxMIS tool uses a relatively small number of predictors (7). Finally, these previous risk prediction studies used variables from structured data fields in the EHR. However, we have found that clinicians tend to record the information used in the OxMIS tool in free-text documents rather than structured fields. We therefore aimed to use NLP approaches to extract this information. Using free-text notes could allow the extraction of different types of variables compared to previous suicide prediction tools (35, 36).

NLP has been used to extract a variety of information from free-text clinical notes, demonstrating a large range of potential uses. These applications include extracting concepts such as drug polypharmacy (17), symptoms of mental illness (37), the presence of suicidal behaviors (20, 38), or employment status (21). These approaches share similarities with ours and support the idea that it is feasible to use NLP to extract historical variables from EHRs. Other studies have used different NLP approaches which do not involve interpretation of the linguistic context within which words appear. One example is assessing the overall positive or negative valence in discharge summaries in relation to risk of suicide and suicide attempts (15, 39), or using a “bag-of-words” approach which analyses text in terms of the frequency of specific words to predict suicide (40), or seclusion for psychiatric inpatients (41). While the ‘bag-of-words’ approach is intuitively clear and serves as a good baseline model, it lacks the ability to capture the contextual information. For example, *“a rabbit ate an apple”* and *“an apple ate a rabbit”* will result in the same collection of tokens (apple, ate, rabbit) whilst it’s obvious to humans that these two sentences are completely different. Recent progress in the field of NLP and language modelling (42), offer novel opportunities for information retrieval from medical records to build on previous work.

#### Strengths, Limitations, and Future Research

One strength of this feasibility study is that we have used a data source which reflects the information available to practitioners who would ultimately use the risk tool. The cohort of patients studied is naturalistic, representing real-world individuals accessing secondary mental health services. The approach explored here has the potential to make it feasible to perform external validation of a risk prediction tool in novel populations which is important for evidence-based decision-making regarding the implementation of such tools.

In addition, the NER tool which we have developed may have many applications within research and clinical practice. The concepts we have extracted (such as history of self-harm and education history) are relevant to researchers in many fields. Further refinements to the model are required, and the generalizability to other EHR systems needs to be considered but the applications are potentially broad.

Two limitations should be noted. EHR data have not been collected for research purposes. As such there may be biases in data collection, and some variables may differ from how they are recorded in data registers. In addition, the threshold for an event to be recorded in the dataset may differ in electronic health records compared to national registers. These differences could have implications for the use of EHRs for external validation studies of tool developed using different data sources. For example, during tool development, self-harm history was extracted from clinical codes in a national register, and therefore represented incidents of self-harm which resulted clinical service contact. Clinical records, on the other hand, may record less severe self-harm which would not have been formally coded. A second difficulty when comparing EHR data with national registers relates to parental variables. During OxMIS tool development, linked parental health records were searched for relevant data such as drug and alcohol use disorder diagnoses. In contrast, with electronic health records data, data availability depends on what questions a clinician has asked a patient and recorded unambiguously in the clinical notes—a process that is subject to bias. A consequence of this is that the variables extracted from electronic health records may differ from variables used in prediction model development. This could influence the performance of the model or result in the external validation testing what is effectively a new model. On the other hand, the data contained within electronic health records is closer to the information available to clinicians, and EHRs provide a source of retrospective data where the accuracy of a risk prediction model can be assessed without confounding effects from altered management decisions due to tool use. It is not clear at present whether variables recorded in EHRs accurately match those contained in population registers, or the implications of any differences for model performance. However, these differences are an important consideration for external validation of a risk assessment tool using EHRs. One further consideration is that if clinicians routinely use a standardized risk assessment such as OxMIS in clinical practice, this might prompt them to gather and record more standardized information in EHRs. This in turn would bring the information in EHRs closer to that in data registers.

Future research into the OxMIS tool will need to further address the feasibility of clinical implementation, this will likely involve incorporation into EHRs using structured data fields rather than natural language processing—the clinician may be asked to record the tool variables within the EHR as a routine part of clinical care. To fully evaluate feasibility it will be important to compare the OxMIS tool to current clinical practice and evaluate its acceptability and utility for clinicians, as has previously been done for similar risk assessment tools in forensic psychiatry (43). In addition, the effects of tool use on clinical care should ultimately be evaluated prospectively, as has been proposed for tools assessing psychosis risk (44). However, the use of NLP within the EHR may constitute a powerful research tool enabling the external validation of the OxMIS tool using a large retrospective cohort. There are several options for further development of our NLP approach which individually or in combination may make it suitable for use in external validation. One approach would be to undertake further manual annotation of clinical records to provide further training data for the model to improve interpretation of linguistic context (such as relation to family members). A second option could be to use the NLP model to screen text for variable information, categorize obvious instances and refer less “certain” instances to a human for arbitration.

## Conclusion

In this feasibility study, we found that clinicians routinely record the predictors used for suicide risk prediction in the OxMIS tool and it was feasible to estimate 12 month suicide risk based on clinical records. However much of this information is recorded in free-text clinical notes. Using machine learning, we developed a named entity recognition model for electronic health records which showed good performance in extracting multiple concepts including medication use, history of violence, and previous self-harm. These findings suggest that natural language processing approaches may facilitate the use of EHRs to study these suicide predictors in populations for which such information was previously unavailable, with applications for external validation and development of risk prediction tools.

## Data Availability Statement

The datasets generated for this study are based on electronic health records which potentially contain identifiable patient information and are therefore not publicly available. Access to CRIS data is restricted to authorized users with approval of the CRIS oversight group. Details can be found at: https://www.oxfordhealth.nhs.uk/research/toolkit/cris/

## Ethics Statement

Study 1 was approved by the Oxford Health NHS Foundation Trust in January 2019 as a Service Evaluation project, therefore ethical approval and informed consent was not required, as per local legislation and national guidelines. Study 2, was approved by the local CRIS oversight committee, and is covered by approval for the CRIS database granted by the Oxfordshire Research Ethics Committee C. Individual patient consent was not required for this use of anonymized, routine data, as per local legislation and national guidelines.

## Author Contributions

SF and RY conceived and designed studies 1 and 2. MB, SP, and JZ were involved in study design for study 1. MS, AK, QL, NV, and AN-H were involved in study design for study 2. MB carried out data extraction and analysis for study 1. MS carried out annotation for study 2 along with Lulu Kane. QL, AK, AN-H, and NV led on NLP model development. MS and AK drafted the manuscript. MS, MB, RY, AK, QL, NV, and SF all critically reviewed the manuscript.

## Funding

This project was funded and supported by the National Institute for Health Research’s Collaboration for Leadership in Applied Health Research at Oxford Health NHS Foundation Trust (NIHR CLAHRC Oxford), now recommissioned as NIHR Applied Research Collaboration Oxford and Thames valley. SF is funded by the Wellcome Trust, grant number 202836/Z/16/Z. AK, NV, QL, and AN-H were supported by the MRC Pathfinder Grant (MC-PC-17215). This work was supported by the UK Clinical Record Interactive Search (UK-CRIS) system using data and systems of the NIHR Oxford Health Biomedical Research Centre (BRC-1215-20005).

## Conflict of Interest

The authors declare that the research was conducted in the absence of any commercial or financial relationships that could be construed as a potential conflict of interest.

The handling editor declared a past collaboration with one of the authors, SF.

## Footnotes

1 https://oxrisk.com/oxmis/

2 https://github.com/explosion/thinc
