# Supplementary material for: Identifying Predictors of Suicide in Severe Mental Illness: A Feasibility Study of a Clinical Prediction Rule (Oxford Mental Illness and Suicide Tool or OxMIS)
Source: Front Psychiatry. 2020 Apr 15;11:268. doi: 10.3389/fpsyt.2020.00268 (PMC7175991; doi:10.3389/fpsyt.2020.00268)
Supplement: Supplementary file 1 [file DataSheet_1.pdf]

Supplemental Figure 1: Structure of annotation schema and named entity recognition model

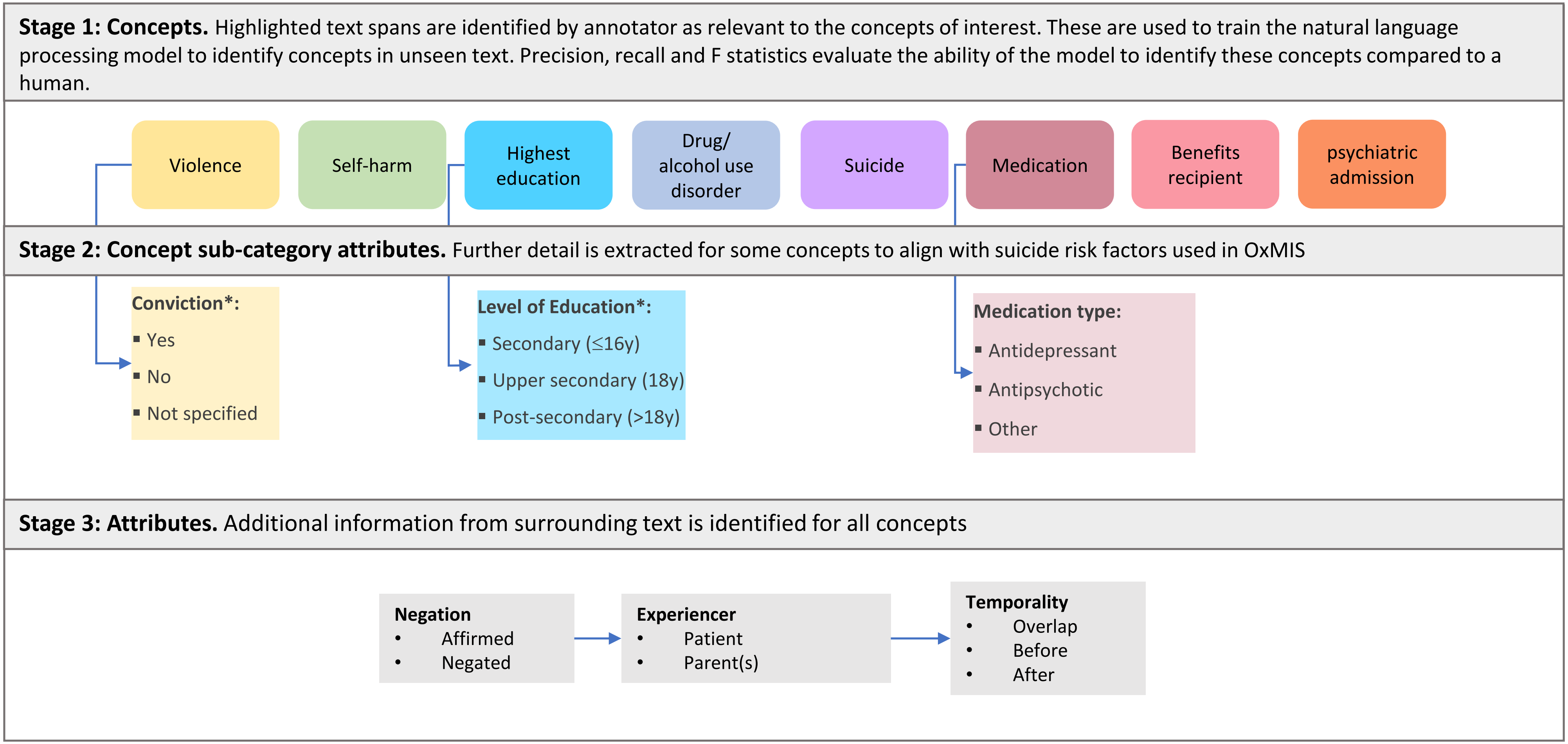

Notes: the steps described above were used to annotate free-text documents from the electronic health record and reflect the structure of the named entity recognition model. Text spans are categorised according to the concept that they describe, and additional sub-categories and attributes are labelled which are necessary to interpret the text as it relates to risk factors for suicide. This study focusses primarily on Stage 1 of the NLP model, and stage 2 for the medication concept.
